# 关注GGO，治疗合理化

**DOI:** 10.3779/j.issn.1009-3419.2018.03.28

**Published:** 2018-03-20

**Authors:** 伦旭 刘, 克能 陈, 树庚 高

年会，又一次的期待如约而至，这不仅是我们广大胸外科医师欢聚的时候，更是我们相互学习、共同进步的时刻。学术内容永远是我们对年会期待中的更期待。虽然今年年会在时间准备上较为仓促，但我们所收到的论文却十分丰厚，共计收到各类投稿300余篇，创历年年会征稿之新高，生动而具体地反映了大家对年会学术日益高涨的期盼和参与热情。我们将一如既往的在能力所及的情况下为大家做好服务工作，尽可能的让更多优秀的稿件在年会召开的时侯，同步刊登在相关的杂志上。遗憾的是，由于受到版面限制，今年年会的许多优秀稿件没能被及时刊出，我们在这里向广大同道们致以深深的歉意。

本次年会杂志所刊出的稿件，一改上届年会以约稿为主的风格，而改由着眼于自由投稿的选用。稿件的选用历经协会组织的专家初审推荐，杂志编辑部再送外审专家复审等许多程序，最终决定是否刊用。除有部分特约专家专题讲座直接刊出外，我们对每一篇原创稿件，都配发了相应领域知名专家的特约述评。希望能对广大的胸外科医师，以及相关专业的读者有所启发。

在此，我们由衷的想感谢赫捷院士在百忙中写就的针对协会，尤其是针对青年医师的谆谆教导。由衷的感谢两位前任会长高屋建瓴的专题报告。由衷的感谢协会的各位副会长、常委、委员，尤其感谢那些并不是委员的大专家们的学术贡献。他们在非常短的时间内倾注的大量心血为此次年会撰稿，成功地写就了一篇篇专题报告，和特约述评。你们的付出将会惠及广大患者，将会对广大胸外科的年轻医生的成长产生深远的影响。在此，我们代表此次年会的主办方及承办方向你们致敬。我们感谢《中国肺癌杂志》为此次年会提供了两期的版面刊发稿件。

第3期的话题，首先，仍然是早期肺癌的诊断与治疗，尤其是我国特殊类型"GGO"样肺癌治疗原则的大讨论。20天前的会前专题讨论会也重点涵盖了这一问题。此次刊用的特约专题，不但有王群教授那次讲座的总结，还有来自肺切除术大户之一的上海肺科医院姜格宁教授团队，有关"GGO"肺癌诊治的医院共识，更有来自医科院肿瘤医院的就这一问题的原创研究。来自安徽、福建等地的原创研究也是精彩纷呈。每篇研究同时配有特邀述评，可以看出大家虽对此问题仍有争议，但基本共识是：需要加强GGO样肺腺癌的研究与质控，避免可能存在的"过度治疗"。这是继上届年会"早期肺癌"专题之后的再讨论、再争议。相信大家都会从中获益。

与此同时，本期更多的关注了早期肺癌治疗后的康复这一话题，包括适应症选择问题、合并疾病的处理、微创手术技术掌握问题、可能致命的手术并发症预防问题，以及术后随访与管理的问题等。目的在于引导大家应严格选择手术适应证，加强技术训练，关注术后并发症处理，及长期生存者的医学及人文关怀。使我们胸外科治疗肺癌更加合理有效，使手术与生俱来的伤害尽可能降到最低。

由于我们水平有限，相信有许多地方没有充分反映一年来大家在行业里的业绩，肯定也有许多不当不到之处，在此敬请大家海涵。我们在接受批评的同时，更期待大家在下次年会的精彩研究。

